# Antihyperglycemic Activity of *Eucalyptus tereticornis* in Insulin-Resistant Cells and a Nutritional Model of Diabetic Mice

**DOI:** 10.1155/2015/418673

**Published:** 2015-08-20

**Authors:** Alis Guillén, Sergio Granados, Kevin Eduardo Rivas, Omar Estrada, Luis Fernando Echeverri, Norman Balcázar

**Affiliations:** ^1^Molecular Genetics Group, University of Antioquia, Calle 70, No. 52-21, A.A. 1226, Medellín, Colombia; ^2^Department of Physiology and Biochemistry, School of Medicine, University of Antioquia, Calle 70, No. 52-21, A.A. 1226, Medellín, Colombia; ^3^Laboratory of Cellular Physiology, Center of Biophysic and Biochemistry, IVIC, Carretera Panamericana km 11, Altos de Pipe, Caracas, Venezuela; ^4^Group of Organic Natural Product Chemistry, Faculty of Natural and Exact Sciences, University of Antioquia, Calle 70, No. 52-21, A.A. 1226, Medellín, Colombia

## Abstract

*Eucalyptus tereticornis* is a plant used in traditional medicine to control diabetes, but this effect has not been proved scientifically. Here, we demonstrated through *in vitro* assays that *E. tereticornis* extracts increase glucose uptake and inhibit their production in insulin-resistant C2C12 and HepG2 cells, respectively. Furthermore, in a nutritional model using diabetic mice, the administration of ethyl acetate extract of *E. tereticornis* reduced fasting glycaemia, improved tolerance to glucose, and reduced resistance to insulin. Likewise, this extract had anti-inflammatory effects in adipose tissue when compared to control diabetic mice. Via bioguided assays and sequential purification of the crude extract, a triterpenoid-rich fraction from ethyl acetate extracts was shown to be responsible for the biological activity. Similarly, we identified the main compound responsible for the antihyperglycemic activity in this extract. This study shows that triterpenes found in *E. tereticornis* extracts act as hypoglycemic/antidiabetic compounds and contribute to the understanding of their use in traditional medicine.

## 1. Introduction

Type 2 diabetes mellitus (T2DM) is characterized because pancreatic *β* cells cannot synthesize adequate amounts of insulin to satisfy the metabolic demand of peripheral tissues such as skeletal muscle, adipose tissue, and liver [1, 2]. There is a strong association between T2DM and obesity; for instance, increase in body mass index (BMI), especially in the abdominal region, is related to increase in insulin resistance and in the risk of developing T2DM [3, 4]. Consequently, obesity is found in 90% of T2DM patients. In obesity, as calorie intake increases, adipocyte hypertrophy increases because of an increase in stored triacylglycerol (TAG) [5]. When the hypertrophy reaches a threshold and remains over time, the endocrine function of adipocytes is altered and a special microenvironment is established. This induces oxidative stress, inflammation, and the release of nonesterified free fatty acids (FFAs), which are phenomena involved in generating insulin resistance (IR), both in adipose tissue and in peripheral organs. It is also the greatest risk factor in developing T2DM.

Currently, there are a variety of T2DM treatments, but the presence of side effects, limited therapeutic effects, and intravenous administration has led to a worldwide search for new and better therapeutic agents [6]. Regarding this issue, traditional medicine is particularly valuable for the development of new treatments with the advantage that information exists about its safe therapeutic effect* in vivo* [7]. Colombia has notable biodiversity and a great cultural tradition in the therapeutic use of plants. Nevertheless, the scientific analysis of these medicinal plants remains unexplored.

Previous studies identified* Eucalyptus globulus* compounds that reduce oxidative stress in diabetic rats [8], and ursolic acid isolated from* E. tereticornis* avoids the accumulation of lipids in hepatic rat cells [9]. In Swiss Webster mice, raw extracts from* E*.* tereticornis* presented antihyperglycemic activity evaluated through an oral glucose tolerance test (OGTT) [10]. The experimental models developed in the studies previously mentioned simulate more type 1 diabetes mellitus than type 2. The latter is more prevalent and accounts for about 90% to 95% of all diagnosed cases of diabetes.

On the other hand, it is relevant to use animal models derived from genetic and environmental factors [11, 12] that simulate type 2 diabetic patients, to study their pathophysiological events and to evaluate the actions or the mechanisms of the new therapeutic agents.

In this study, we investigated the effect of* E. tereticornis* on insulin sensitivity in mice and in HepG2 and C2C12 insulin-resistant cell lines. Streptozotocin (STZ) treated and high-fat diet-fed C57BL/6J mice were selected as diabetic animal models because of their close similarities to type 2 diabetic patients. Effectively, extracts and a pure compound from* E. tereticornis* displayed an antihyperglycemic effect in both* in vitro* and* in vivo* assays.

## 2. Materials and Methods

### 2.1. Chemical and Reagents

All solvents used for extraction and fractionations, methanol, ethanol, ethyl acetate,* n*-hexane, and dichloromethane, were previously distilled from commercial sources. Thin layer chromatography (TLC) was run in aluminum-backed F_254_ silica gel chromatoplates (Merck, Darmstadt, Germany). Column chromatography was performed with Sephadex LH-20 (Sigma, St. Louis, MO, USA) and glass columns (60 × 7 cm) filled with silica gel 60H (Merck) as well as with glass columns (40 × 3 cm). ^1^H and ^13^C-NMR and two-dimensional spectra were obtained in an AMX 300 spectrometer (Bruker BioSpin GmbH, Rheinstetten, Germany) operating at 300 MHz for ^1^H and 75.0 for ^13^C using CDCl_3_ or DMSO d_6_. Shifts are reported in *δ* units (ppm) and coupling constants (*J*) in Hz.

PBS 1x (Gibco, Carlsbad, CA, USA), low glucose Dulbecco's Modified Eagle Medium (DMEM), fetal bovine serum (FBS), penicillin-streptomycin (P/S) 10,000 U/mL, glutamine, bovine serum albumin (BSA), sodium lactate, sodium pyruvate, sodium bicarbonate, sodium palmitate and streptozotocin (STZ, Sigma, St. Louis, MO, USA), and 100 IU/mL insulin were used.

Plasma glucose level was measured using a commercial glucose oxidase-peroxidase kit (BioSystems, Bogotá DC, Colombia); plasma insulin level was measured using an enzyme-linked immunosorbent assay (ELISA) kit (Mercodia AB, Uppsala, Sweden); viability of the cells was measured by methyl thiazole tetrazolium (MTT, Amresco, Solon, OH, USA) assay. All measurements were performed using a Varioskan Flash spectrophotometer (Thermo, Waltham, MA, USA) at 500 or 570 nm. Glycaemia in mice was measured with the GlucoQuick Glucometer (Procaps, Barranquilla, Colombia).

### 2.2. Preparation of the Extract and Chromatographic Fractionation

*E. tereticornis* leaves were collected in Valledupar (Colombia) in 2011. A specimen was deposited in the Herbarium of the University of Antioquia with # 178511. The dried leaves (2 kg) were extracted with 2 L 96% methanol at room temperature for 24 h and then filtered and concentrated to dryness at 38°C under reduced pressure to yield 120 g of crude methanol extract (crude extract). This extract was dissolved in 250 mL of a water-methanol mixture (1 : 1, v/v) and then partitioned with 400 mL (4 × 100 mL) petroleum ether and subsequently with 400 mL (4 × 100 mL) ethyl acetate, to afford to produce extracts F1 (48.0 g) and F2 (17.0 g), respectively.

Subsequently, 4.0 g ethyl acetate extract (from now on, designated F2) was fractionated on a Sephadex LH-20 column using hexane-dichloromethane-methanol 2 : 1 : 1 (v/v) as eluent and monitoring by silica gel TLC in hexane-ethyl acetate 1 : 1 (v/v); eight fractions were collected (F2-1 to F2-8), which were analyzed for the* in vitro* glucose uptake. Fractions F2-3 and F2-4 showed similar compositions, but the latter fraction (300 mg) contained several triterpenes, as revealed by TLC, and provoked high hypoglycemia activity.

The compounds contained in the active fraction F2-4 (300 mg) were separated on a silica gel column, eluted with a hexane-ethyl acetate 9 : 1 (v/v) mixture, which increased in polarity to finish with pure ethyl acetate; a total of 26 fractions were collected. From this new column, the* in vitro* active fractions 21 and 26 (F2-21 and F2-26) showed the presence of three compounds by TLC in hexane-ethyl acetate (1 : 1, v/v) that were separated by preparative chromatography in the same system (three runs). In this way, 20 mg of the active compound** 1** with hypoglycemic activity was obtained. The other two substances could not be identified due to low yield and complexity of the NMR spectra.

### 2.3. Cell Cultures

The C2C12 (ATCCCRL-1772) mouse muscle cells and HepG2 (ATCC HB-8065) human liver carcinoma cells were purchased from ATCC (Manassas, VA, USA). The cells were cultured and maintained at 37°C and 5% CO_2_ in DMEM culture medium with 10% fetal bovine serum (FBS), 2 mM glutamine, penicillin, and 1% streptomycin (Sigma-Aldrich, St. Louis, MO, USA). When the C2C12 cells reached 80 to 90% confluence, they were differentiated into myotubes, using low glucose DMEM (5.5 mM) supplemented with 5% horse serum (HS) [13].

### 2.4. Insulin-Resistant Cell Model

To develop a model of insulin-resistant cells, 4-day differentiated myotubes and HepG2 cells were preincubated for 2 h in DMEM with 5.5 mM glucose without FBS and supplemented with 1% BSA and then incubated for 18 h in DMEM with 5.5 mM glucose without FBS, 1% BSA, 0.75 mM palmitate, and 200 mM insulin [13, 14].

C2C12 myotubes were incubated for 4 h in 500 *μ*L DMEM, 5.5 mM glucose, and various chromatographic fractions or extracts. After 4 h of incubation, 500 *μ*L of supernatant was collected, and glucose concentration was measured using the glucose oxidase/peroxidase technique with a commercial kit. To calculate glucose utilization, the remaining glucose in the culture medium after incubation with controls and extracts or fractions was subtracted from the initial amount of glucose (5.5 mM).

HepG2 cells were washed three times with PBS to remove glucose, incubated for 4 h in 1 mL glucose production medium (glucose and phenol red-free DMEM supplemented with 20 mM sodium lactate and 2 mM sodium pyruvate) and in the presence of 1 nM insulin during the last 3 h. Medium (300 *μ*L) was sampled to measure glucose concentration using the Amplex Red Glucose/Glucose Oxidase Assay Kit (Invitrogen, MA, USA). Commercial metformin (100 mg; Laboratorio Memphis, Bogotá DC, Colombia) was pulverized, dissolved in cultured media, and used as positive control in both cell lines.

### 2.5. Glucose and Insulin Tolerance Test in the Mouse Model of T2DM

C57BL/6J male mice (Charles Rivers Laboratories, Wilmington, MA, USA) over 4 weeks old were used in this study. The mice were housed at 22 ± 2°C with a 12 : 12 h light dark cycle with free access to food and water for 8 weeks. They were then randomly divided into three groups. A control group (*n* = 10) was fed a normal diet (ND, 14% fat/54% carbohydrates/32% protein). A high-fat diet (HFD) + STZ group (*n* = 10) and an HFD + STZ + F2 group (*n* = 10) were fed a high-fat diet (HFD, 42% fat, 42% carbohydrates, and 15% proteins) and received 3 doses of STZ at a low concentration (25 mg/kg) at the same time every day, after 4-hour fasting. The HFD + STZ + F2 group received 10 intraperitoneal doses, one dose every two days of F2 300 mg/kg. All animal studies were approved by the Institutional Animal Care and Use Committee of the University of Antioquia (Protocol number 65). Before and after treatment with F2, an intraperitoneal glucose tolerance test (IPGTT) was performed on the mice by administering a glucose load of 2.0 g/kg body weights. An insulin tolerance test (ITT) was performed after overnight fast. Initial blood glucose levels were determined, followed by injection (IP) of human insulin 0.75 U/kg. Blood glucose levels were measured via tail vein blood at 0, 30, 60, and 120 min after the injection using a GlucoQuick Glucometer (Procaps, Barranquilla, Colombia). Zero time was measured just before glucose injection. After glucose metabolism studies, mice were sacrificed and blood, liver, and adipose tissue were collected for further analyses.

### 2.6. RNA Extraction and Real-Time PCR

Total RNA was extracted from liver and visceral white adipose tissue with the RNeasy kit (QIAGEN, Valencia, CA), and the reverse transcription reaction was performed with 500 ng total RNA, 50 ng/*μ*L random hexamers, 10 mM dNTP Mix, 20 mM Tris-HCl pH 8.4, 50 nM KCl, 2.5 mM MgCl_2_, 40 U/*μ*L RNaseOut, and 200 U/*μ*L SuperScript III RT (Invitrogen, MA, USA) according to the manufacturers' instructions.

Real-time quantitative PCR (qPCR) analyses were performed with 50 ng cDNA and 100 nM sense and antisense primers (Integrated DNA Technologies, Coralville, IA, USA) in a final reaction volume of 25 *μ*L using the Maxima SYBR Green/ROX qPCR Master Mix (Thermo Scientific, Waltham, MA, USA) and the CFX96 real-time PCR detection system (Bio-Rad, Hercules, CA, USA). Specific primer sequences are provided in supporting information Table 1  (see Supplementary Material available online at http://dx.doi.org/10.1155/2015/418673). Relative quantification of each gene was calculated after normalization to GAPDH RNA by using the comparative CT method. The program for thermal cycling was 10 min at 95°C, followed by 40 cycles of 15 s at 95°C, 30 s at 60°C, and 30 s at 72°C.

### 2.7. Statistical Analysis

The results are expressed as means ± SEM. Student* t*-tests were used to compute individual pairwise comparisons of least square means. The trapezoidal rule was used to determine the area under the curve (AUC). Differences were considered to be significant at *p* < 0.05. All analyses were performed with the Prism 4 (GraphPad software Inc., La Jolla, CA, USA) statistical software.

## 3. Results

### 3.1. Hypoglycemic Effect of F2 on C2C12 and HepG2 Insulin-Resistant Cells

**Figure 1:**
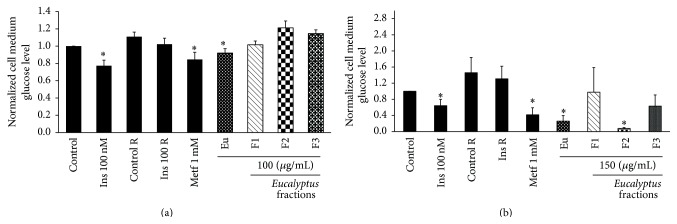
Effect of raw extract and fractions from* E. tereticornis* on insulin-resistant C2C12 and HepG2 cells. Glucose uptake in insulin-resistant C2C12 cells (a) and glucose production in insulin-resistant HepG2 cells (b). Cells were treated with different concentrations of the fractions or raw extract and glucose was measured in cultured supernatant after 4 h of treatment by the glucose oxidase technique. Eu, crude methanol extract; Ins (100 nM insulin); Control R, insulin-resistant C2C12 myotubes as controls; Ins 100 R, insulin-resistant C2C12 myotubes treated with 100 nM insulin; Metf, metformin 1 mM positive control. Bar values correspond to the arithmetic mean of glucose concentration for each treatment/control glucose concentration, *n* = 6 (C2C12) and *n* = 3 (HepG2). ^**∗**^Ins 100 nM versus control. ^*∗*^Treatment versus control R. *p* < 0.05, Student* t*-test. Error bars represent SEM.

Figure 1 shows the effect of crude methanol extract and different extracts, F1, F2, and F3, on glucose uptake and production in cells previously exposed to palmitate.  Figure 1(a) demonstrates that the glucose concentration in response to insulin in cells pretreated with palmitate was reduced by 14.8% compared with its control (Control R). Comparatively, when cells had not been treated previously with palmitate and were exposed to insulin (Ins 100 nM), the reduction of glucose concentration in the supernatant compared to its control (Control) was 23%. This means that palmitate induced a 35.7% reduction of the effect of insulin showing that C2C12 cells were insulin resistant. Likewise, under conditions of insulin resistance, the effect of crude extract (100 *μ*g/mL) and its different extracts (gray bars in Figure 1(a)) on glucose uptake was determined. After testing various crude extract concentrations (data not shown), it was determined that there was a greater increase of glucose uptake at 100 *μ*g/mL and that it was 17.1% in comparison with its control (Control R). Nevertheless, this response was lower than the one obtained with a positive control (Metf 1 mM). Regarding the fraction involved in response to the extract, F2 treatment showed a similar glucose uptake compared to the extract. No significant difference was found between this condition and the control (Control R).

On the other hand, the inhibition of glucose production in response to insulin was measured in control conditions using HepG2 cells pretreated with palmitate.  Figure 1(b) shows a 36% inhibition of glucose production when insulin is present (Ins 100 nM) in comparison with control cells. Nevertheless, when the cells were pretreated with palmitate and stimulated with insulin (Ins R), this inhibition was reduced to 7% when compared with its control (Control R) indicating a state of resistance to the hormone. The effect of crude extract with its respective solvent extracts F1, F2, and F3 (gray bars in Figure 1(b)) was evaluated for 4 h in insulin-resistant cells. Crude extract at 150 *μ*g/mL inhibited 82% of the glucose production in comparison with its control (Control R, Figure 1(b)). This value was greater than the percentage of inhibition obtained with the positive control (Metf 1 mM). It is also shown that the fraction involved in the response obtained with the extract is F2 with a 95% inhibition in comparison with its control (Control R).

### 3.2. F2 Improved Carbohydrate Metabolism in the Type 2 Diabetes Mouse Model

**Figure 2:**
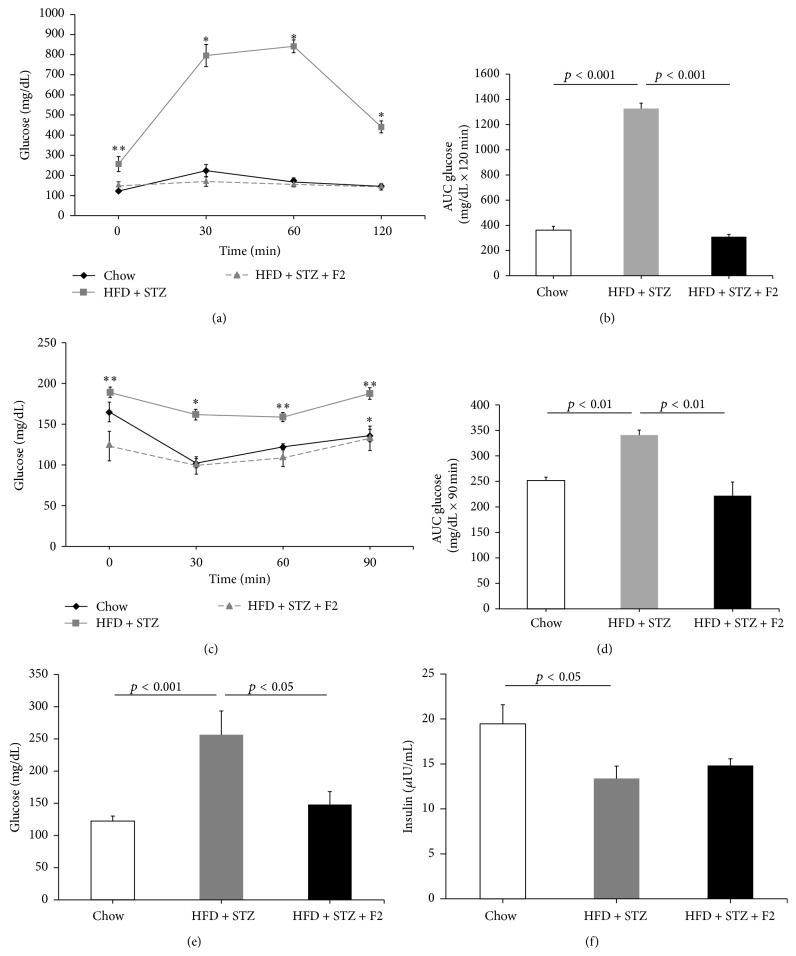
Glucose tolerance test (IPGTT) (a), insulin tolerance test (ITT) (c), fasting blood glucose levels (e), and fasting blood insulin levels (f) in F2 treated diabetic mice. All assays were performed after 10 IP treatment doses. ((b) and (d)) Area under the curve (AUC) values, calculated using data obtained in (a) and (c). HFD + STZ: diabetic control group and HFD + STZ + F2: diabetic group given 10 IP doses of ethyl acetate fraction (F2). (*n* = 6.) ^*∗*^ *p* < 0.05 and ^*∗∗*^ *p* < 0.001, Student* t*-test. Values are expressed as mean ± SEM.

To determine glucose tolerance and insulin resistance of diabetic mice and diabetic mice treated with F2, IPGTT and ITT were conducted. The results were analyzed as the total area under the curve (AUC) between 0 and 120 min or 0 and 90 min in Figures 2(a) and 2(c), respectively. The results obtained in the IPGTT (Figures 2(a) and 2(b)) show a decrease in glucose tolerance in diabetic mice (HFD + STZ) in comparison with the controls (normal diet). Treatment with F2 (HFD + STZ + F2) significantly increased glucose tolerance (Figures 2(a) and 2(b)). The ITT showed that treatment with HFD + STZ caused insulin resistance and that it is reverted when mice were treated with F2 (Figures 2(c) and 2(d)).

The fasting glucose concentration in the three animal groups of the study is presented in Figure 2(e). Diabetic mice (HFD + STZ) have glycaemia almost twice as high as the controls (normal diet). F2 treatment reverted fasting hyperglycemia.  Figure 2(f) shows plasma insulin concentration; insulin level decreases in the diabetic group in comparison with the control, just as expected. Nevertheless, this value did not increase in the group treated with F2 (Figure 2(e)).

### 3.3. F2 Reduced Proinflammatory Cytokine mRNA Expression in Adipose Tissue from Diabetic Mice

**Figure 3:**
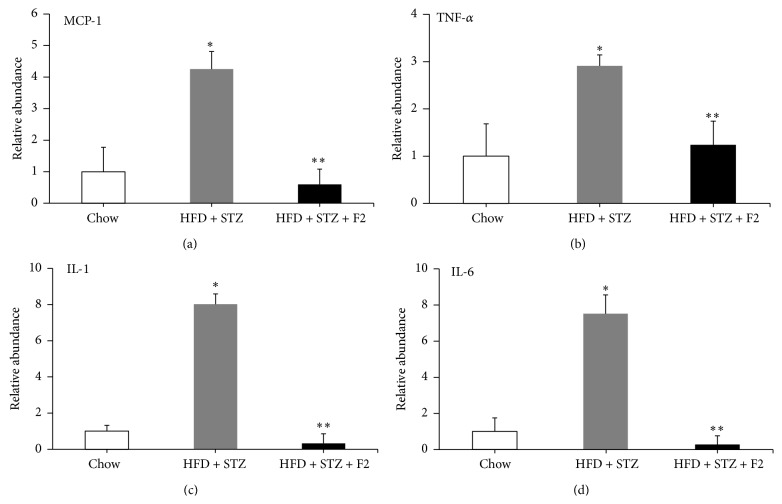
Effect of F2 on adipose expression of proinflammatory cytokine genes. mRNA expression levels by qRT-PCR of MCP-1 (a), TNF-*α* (b), IL-1*β* (c), and IL-6 (d) in adipose tissues of F2 treated diabetic mice. HFD + STZ: diabetic control group and HFD + STZ + F2: diabetic group given 10 IP doses of ethyl acetate fraction (F2). (*n* = 6.) ^**∗**^ *p* < 0.05 versus control and ^**∗∗**^ *p* < 0.05 versus HFD + STZ group. Values are expressed as mean ± SEM.

To determine if F2 improved the inflammation process caused by the HFD diet, we evaluated the mRNA expression of four proinflammatory cytokines, MCP-1, TNF-*α*, IL-1, and IL-6, in adipose tissue of diabetic mice (HFD + STZ) and diabetic mice treated with F2 (HFD + STZ + F2) including their respective controls. In Figure 3, we observe a significant increase, from 3 to 8 times, of the expression of the four cytokines in diabetic mice in comparison with their respective controls. This alteration was reverted in mice that received 10 doses of F2.

### 3.4. F2 Reduced Glucose-6-Phosphatase (G6Pase) mRNA Expression in Liver from Diabetic Mice

**Figure 4:**
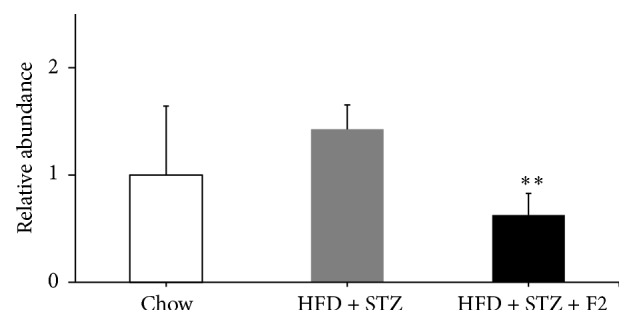
Effect of F2 on hepatic gluconeogenic gene. mRNA expression levels by qRT-PCR of glucose-6-phosphatase (G6P) in hepatic tissue of ethyl acetate fraction (F2) treated diabetic mice. HFD + STZ: diabetic control group and HFD + STZ + F2: diabetic group given 10 IP doses of F2. (*n* = 6.) ^**∗∗**^ *p* < 0.05 versus HFD + STZ group. Values are expressed as mean ± SEM.

To evaluate the influence of F2 on gluconeogenesis, we evaluated the expression of the G6Pase gene in the liver of mice of the three study groups. In Figure 4, a rising trend (*p* = 0.06) of G6Pase expression in diabetic mice is visible in comparison with mice fed a normal diet; nevertheless, this increase was significantly reduced to 37.7% in diabetic mice treated with F2.

### 3.5. Structure of Compound** 1** with Hypoglycemic Effects

The ^1^H NMR spectra (300 MHz, CDCl_3_) showed signs of methyl groups at *δ* 0.83 (s), 0.95 (d, *J* = 6.0), 1.03 (s), 1.05 (d, *J* = 6.0), 1.10 (s, 3H), 1.20 (s), and 1.29 (s) and a dd in 3.25 (1H), a triplet in 4.26 (1H, disappeared adding D_2_O), a dd (1H, *J* = 3.0 y 10.0) in 5.75, and doublet (1H, *J* = 10.0).

^13^C RMN (DEPT 135, 75.0 MHz, and CDCl_3_) spectrum showed the following signals, *δ* 14.95 (q), 16.13 (q), 17.70 (t), 17.85 (q), 17.93 (q), 18.91 (q), 19.19 (q), 22.82 (t), 25.55 (t), 27.02 (t), and 27.79 (q), and the following doublets, 30.83, 31.23, 31.33, 38.14, 38.30, 40.27, 53.04, 54.76, 60.58, 78.85, 128.82, and 133.50. Moreover, there were signals of quaternary carbons (singlets) at *δ* 36.37, 38.02, 38.80, 38.94, 40.02, 41.70, 41.95, 89.75, and 180.00.

**Figure 5:**
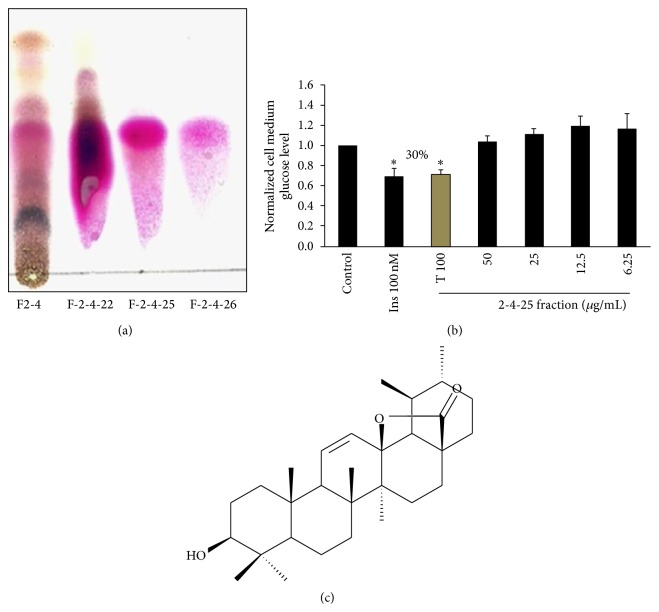
Effect of the fraction 2-4-25 enriched in compound** 1** on glucose uptake in C2C12 cells. (a) Thin-layer chromatography. Fraction F2-4 was separated again on a silica gel column. Subfraction 22 shows a large amount of triterpenes; pure compound** 1** obtained from subfractions 25 and 26 after TLC. (b) 2-4-25 fraction rich in compound** 1** increases glucose uptake. *n* = 3. ^*∗*^ *p* < 0.05 versus control, Student* t*-test. (c) Structure of compound** 1**: 3*β*-hydroxy-urs-11-en-28,13*β*-olide.

Through experiments, COSY ^1^H-^1^H, HMQC, and HMBC, the complete structure of bioactive compound** 1** was assigned as shown in Figure 5(c) and corresponds to 3*β*-hydroxy-urs-11-en-28,13*β*-olide. This compound was previously described from this plant [15].

## 4. Discussion

In this study, we evaluated the effect of the methanolic extract from* E. tereticornis* leaves and ethyl acetate extract (F2) in two* in vitro* models (C2C12 and HepG2 insulin-resistant cell lines) and an* in vivo* model (C57BL/6J diabetic mice). Our findings can be summarized as follows. (1) Crude extract increased glucose uptake in C2C12 cells and inhibited glucose production in HepG2 cells, both insulin-resistant cells. (2) F2 improved glucose and insulin tolerance in a type 2 diabetes mouse model. (3) F2 had anti-inflammatory activity when evaluated on the adipose tissue of obese mice. (4) Identification of an F2 molecule could be responsible for the activity in this extract in C2C12 cells.

In mammals, skeletal muscle represents approximately 70% of their body mass, and it is the main tissue implicated in insulin-stimulated glucose uptake. It has been well established that muscle glucose uptake is reduced in T2DM [16]. Here we used a C2C12 mouse cell line that produces myotubes that have been used as a skeletal muscle glucose uptake model [17, 18].

One of the relevant aspects of this study is the establishment of models of insulin-resistant myotubes and hepatic cells. This implies a decrease in muscle glucose uptake and a decrease in hepatic gluconeogenesis inhibition. Moreover, in T2DM, low glycogen synthesis and high gluconeogenesis and glycogenolysis are important mechanisms responsible for fasting hyperglycemia [19].

Crude extract increased glucose uptake by 17% in comparison with an insulin-resistant control (Figure 1(a)). However, the uptake percentage was lower than the one obtained from normal cells (23% versus 17%, see supplementary Figure S1), showing that the signaling pathways involved in glucose uptake have been impaired by the chronic action of palmitate. On the other hand, glucose production was evaluated in the HepG2 cell line. Insulin inhibited gluconeogenesis in comparison with controls and the addition of palmitate impaired this response (36% control versus 7% resistance), showing that the cells were insulin resistant (Figure 1(b)).

Crude extract reverted the effect of insulin resistance induced by palmitate by 82% in comparison with resistance control. As for the different solvent extracts, F2 was the fraction involved in such response, obtaining even a greater percentage of inhibition (95%) than the one obtained with pure extract. This demonstrated that the molecule or molecules involved in inhibition of glucose production in the* in vitro* system were mostly located in the F2. In order to validate the results obtained* in vitro*, we decided to evaluate the effect of F2, in a T2DM murine model.

Here, we used a T2DM nutritional model developed on C57BL/6J mice. Taking into account the etiology of the disease caused by a combination of multiple factors, we integrated an environmental component (mice fed with a high-fat diet) with the impairment of a mice's capability to secrete insulin by pancreatic *β* cells using multiple doses at a low STZ concentration, which produces DNA alkylation and increases ROS (reactive oxygen species) production. The latter could facilitate an increase of the glucolipotoxicity in the pancreatic cells of mice fed a HFD. Furthermore, it has been reported that low doses of STZ induce low percentages of *β* cell destruction as observed in T2DM [20, 21]. This is a great difference when comparing this model to others used in which a sole dose is given at a high STZ concentration (100–200 mg/kg) or alloxan (65–150 mg/kg) producing the total destruction of *β* cells, which is characteristic of type 1 diabetes [21, 22].

As expected, this diabetic mice model showed an increase in fasting glucose in comparison to control mice (Figure 2(e)). After administering 10 IP doses of F2, a clear decrease in fasting glucose levels (Figure 2(e)) and an increase in glucose tolerance (Figures 2(a) and 2(b)) were observed. In other words, mechanisms related to the maintenance of glucose homeostasis are most effective because there is greater glucose uptake by peripheral tissues or a lower glucose hepatic production. In mice treated with F2, we also observed a reversion of insulin resistance (Figure 2(d)). Likewise, the molecules present in F2 could be acting in the liver activating routes that favor the inhibition of glucose production, as an insulin pathway, or activating proteins such as AMPK (5′ adenosine monophosphate-activated protein kinase) that inhibit the expression of key genes of gluconeogenesis as in the case of G6Pase. We confirmed that F2 treatment leads to a significant decrease in the expression of the G6Pase gene in liver (Figure 4). Jung et al. (2012) [23] observed a similar mechanism in a study where diabetic mice fed with a diet supplemented with persimmon (*Diospyros *spp.) extract showed lower glycaemia, lipaemia, and less fat accumulation related to a decrease in G6Pase activity.

On the other hand, it is important to highlight the fact that there was a significant decrease in the insulin level in fasting diabetic mice in comparison with controls (Figure 2(f)), indicating pancreatic *β* cell damage of this group. Since the group treated with 10 IP doses of F2 did not increase plasma insulin levels, it is possible that the compounds of F2 did not act in the pancreas or that treatment was insufficient to observe an improvement or an increase of *β* cells that could reflect an increase in insulin levels. These results agree with the ones obtained* in vitro*, where there was no evidence of insulin secretion stimulated by glucose, in MIN6 cells (mouse insulinoma cell line) treated with an F2 (data not shown).

In T2DM, an increase in the expression of proinflammatory cytokines such as TNF-*α* (tumor necrosis factor), IL-6 (interleukin-6), IL-1 (interleukin-1), and MCP-1 chemokine (monocyte chemotactic protein 1) has been demonstrated [24]. MCP-1 is secreted by preadipocytes and adipocytes and participates in recruiting monocytes and macrophages implicated in a chronic low inflammation condition observed in obesity. Once the macrophages have been infiltrated in adipose tissue, they can secrete cytokines that favor the development and/or the deterioration of insulin resistance. The TNF-*α* gene is expressed constitutively in adipose tissue where its expression increases principally due to the infiltration of macrophages. mRNA expression in adipose tissue of the TNF-*α* of obese subjects is 2.5 times greater than in subjects with normal weight, and this high expression level is greatly correlated with hyperinsulinemia. It is an important event in the physiopathology of insulin resistance and T2DM [24, 25].

We evaluated the expression of 4 proinflammatory cytokines in the adipose tissue of the three groups used in the murine model. In Figure 3, an increase in MCP-1, TNF-*α*, IL-1, and IL-6 expression of diabetic mice (fed simultaneously with HFD + STZ) is visible in comparison with controls (normal diet); we clearly noticed that treatment with F2 (HFD + STZ + F2) reduced mRNA levels of all the cytokines. This suggests that F2 compounds reduce the proinflammatory condition of adipose tissue in diabetic mice.

According to the NMR spectra, the ethyl acetate extract (F2) is rich in triterpenes. These molecules are secondary metabolites found in plants distributed around the world. Phytochemical researches have implicated triterpenes as one of the most important chemical groups in which biological activity lies [26].* In vitro* and* in vivo* studies have demonstrated the potential use of these types of molecules (e.g., lupeol, betulinic, ursolic, and oleanolic acids) to prevent and treat a wide spectrum of diseases including cancer, infectious diseases, diabetes, and cardiovascular and rheumatic diseases [27, 28]. Interestingly, the triterpenes studied so far have demonstrated an immunomodulatory activity, which turns them into very promising molecules for treatment of type 2 diabetes [28].

Lupeol is found in various edible vegetable and fruit species as olives, mango, pear, ginseng oil, red fruits, carrots, peppers, tomatoes, guavas, and tea among others. It has been reported that, among all the properties related to these types of molecules, lupeol modulates the expression of various proinflammatory molecules including TNF-*α*, IL-1*β*, prostaglandin E2 (PGE2), IL-2, IL-4, IL-6, and myeloperoxidase [29]. Oleanolic acid (OA) is a pentacyclic triterpene found in a large variety of plants that has a very wide range of pharmacological and biochemical properties including anti-inflammatory, antioxidant, antihyperlipidemic, and hypoglycemic effects [30, 31]. OA reduces insulin resistance related to obesity and hyperlipidemia and protects against endothelial dysfunction [32, 33]. Studies by de Melo et al. (2010) [34] reported that administering 50 mg/dL OA daily to animals fed a high-fat diet significantly reduces abdominal fat making it a candidate to evaluate its anti-inflammatory and antioxidant properties in adipose tissue of obese animals.

Due to the fact that the ethyl acetate extract F2 showed high* in vitro* and* in vivo* activity, it was further fractionated by Sephadex LH-20 column chromatography using a mixture of three eluents to yield eight fractions (F2-1 to F2-8). These fractions were then tested in C2C12 cells using the same methodology as the previous glucose uptake assays. Fraction F2-4 was further fractionated by silica gel column. Preliminary results with fraction F2-4-25 (Figure 5(a)), rich in compound** 1** in glucose uptake in C2C12 cells, suggest that this molecule is involved in* E. tereticornis* hypoglycemia effects (Figure 5(b)). The structure shown in Figure 5(c) corresponds to 3*β*-hydroxy-urs-11-en-28,13*β*-olide, a molecule previously described in* E. tereticornis* with antimicrobial activity [15] and in* E. camaldulensis,* showing an antiproliferative effect on ovarian cancer lines [35]. Despite the large number of pharmacological actions that are attributed to* Eucalyptus *spp., among them antidiabetic, this is the first time we determine one of the potential compounds responsible for such activity.

On the other hand, this molecule is related structurally to other triterpene glycosides isolated from* Boussingaultia baselloides* (syn.* Anredera baselloides)* [36] that have intense hypoglycemic activity and, as such, are used in traditional medicine. Likewise, the treatment with extracts of a plant of the same genus,* Boussingaultia gracilis* Miers var.* pseudobaselloides,* can regulate the expression of genes involved in lipogenesis and lipolysis [37].

Finally, in this study, we demonstrate that F2 reduces the expression levels of proinflammatory cytokines in adipose tissue. This allows us to suggest that the systemic effects of this fraction on the metabolism of carbohydrates could be due to its action in an inflammation established in adipose tissue of mice with obesity-related T2DM. Now we aim to identify the action mechanisms of plant extracts and their active molecules to be able to contribute to the development of new therapeutic strategies that cut the connection between inflammation and oxidative stress and the development of important pathologies in public health such as T2DM. In addition, the next step is to clarify the action mechanisms of compound** 1** in the liver, muscles, and adipose tissue and to determine effects of triterpene mixtures as well as possible synergism among them.

## 5. Conclusions

*E. tereticornis* has potential as hypoglycemic when it is used in insulin-resistant cell models. Furthermore, F2 reduces the expression of proinflammatory cytokines in a T2DM mouse model, which could contribute to improving the cell-signaling pathways in target organs such as muscles and the liver. It results in an increase in glucose tolerance and an improvement of insulin resistance. 3*β*-Hydroxy-urs-11-en-28,13*β*-olide is one of the main molecules of the bioactive F2 extract and is responsible for the antihyperglycemic effects.

## Supplementary Material

Supporting Table 1: Sequences of oligonucleotide primer used for qRT-PCR. All primer sets listed were run for 40 cycles at an annealing temperature of 60 °C. Supporting figure S1. Effect of crude extract and Ethyl acetate extract F2 from E. tereticornis on C2C12 cells. Cells were treated with different concentrations of F2 fractions or crude extract and glucose was measured in cultured supernatant after 4 h of treatment by the glucose oxidase technique. Bar values correspond to the arithmetic mean of glucose concentration for each treatment/control glucose concentration, n=5 (C2C12). ∗Ins 100 nM or treatment vs. control. p< 0,05, t-test. Error bars represent SEM.
