# Supplementary material for: Antihyperglycemic Activity of Eucalyptus tereticornis in Insulin-Resistant Cells and a Nutritional Model of Diabetic Mice
Source: Adv Pharmacol Sci. 2015 Aug 20;2015:418673. doi: 10.1155/2015/418673 (PMC4558436; doi:10.1155/2015/418673)
Supplement: Supplementary file 1 — Supporting Table 1: Sequences of oligonucleotide primer used for qRT-PCR. All primer sets listed were run for 40 cycles at an annealing temperature of 60 °C. Supporting figure S1. Effect of crude extract and Ethyl acetate extract F2 from E. tereticornis on C2C12 cells. Cells were treated with different concentrations of F2 fractions or crude extract and glucose was measured in cultured supernatant after 4 h of treatment by the glucose oxidase technique. Bar values correspond to the arithmetic mean of glucose concentration for each treatment/control glucose concentration, n=5 (C2C12). ∗Ins 100 nM or treatment vs. control. p< 0,05, t-test. Error bars represent SEM. [file 418673.f1.pdf]

Supporting figure S1. Effect of crude and solvent extracts on glucose Uptake using cultured C_2C_{12} Myotubes.

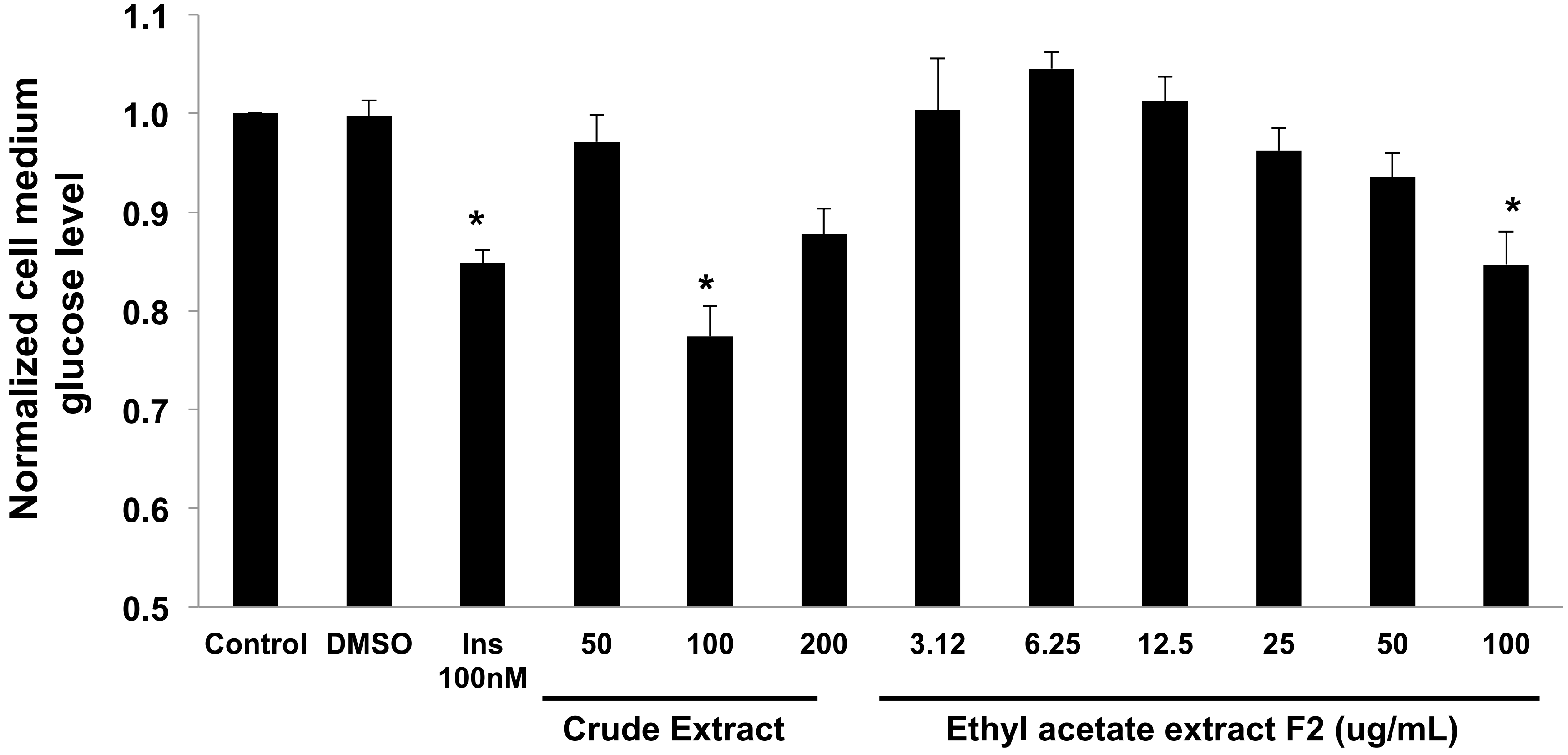

Supporting Table 1.

Primers used for qRT-PCR

Gene	PRIMERS
**G6Pasa**	Forward: TTACCAAGACTCCCAGGACTG
	Reverse: GAGCTGTTGCTGTAGTAGTCG
**MCP-1**	Forward: GCATCCACGTGTTGGCTCA
	Reverse: CTCCAGCCTACTCATTGGGATCA
**TNF-α**	Forward: GCCTCTTCTCATTCCTGCTTG
	Reverse: CTGATGAGAGGGAGGCCATT
**IL-1β**	Forward: CAACCAACAAGTGATATTCTCCATG
	Reverse: GATCCACACTCTCCAGCTGCA
**IL-6**	Forward: GAGGATACCACTCCCAACAGACC
	Reverse: AAGTGCATCATCGTTGTTCATACA
**GAPDH**	Forward: TCACCACCATGGAGAAGGC
	Reverse: GCTAAGCAGTTGGTGGTGCA
